# Supplementary material for: Lithography-free IR polarization converters via orthogonal in-plane phonons in α-MoO3 flakes
Source: Nat Commun. 2020 Nov 13;11:5771. doi: 10.1038/s41467-020-19499-x (PMC7666183; doi:10.1038/s41467-020-19499-x)
Supplement: Supplementary file 1 — Supplementary Information [file 41467_2020_19499_MOESM1_ESM.pdf]

Supplementary information

Lithography-free IR polarization converters via orthogonal in-plane phonons in α-MoO_3 flakes

Sina Abedini Dereshgi[1], Thomas G. Folland[2,3], Akshay A. Murthy[4,5], Xianglian Song[1,6], Ibrahim Tanriover[1], Vinayak P. Dravid[4,5,7], Joshua D. Caldwell[2] and Koray Aydin[1]

[1] Department of Electrical and Computer Engineering, Northwestern University, Evanston, Illinois 60208, United States

[2] Department of Mechanical Engineering, Vanderbilt University, Nashville, Tennessee 37212, USA

[3] Department of Physics and Astronomy, The University of Iowa, Iowa City, Iowa 52242, USA

[4] Department of Materials Science and Engineering, Northwestern University, Evanston, Illinois 60208, USA

[5] International Institute for Nanotechnology, Northwestern University, Evanston, Illinois 60208, USA

[6] International Collaborative Laboratory of 2D Materials for Optoelectronic Science & Technology of Ministry of Education, Engineering Technology Research Center for 2D material Information Function Devices and Systems of Guangdong Province, College of Optoelectronic Engineering, Shenzhen University, Shenzhen 518060, China

[7] Northwestern University Atomic and Nanoscale Characterization Experimental (NU*ANCE*) Center, Northwestern University, Evanston, Illinois 60208, USA

Note 1. TMM and Fabry-Perot analysis.

In order to better understand the mode coupling results of Fig. 2 of the manuscript, transfer matrix method (TMM) and mode analysis results are presented in this section. The structure represented in Fig. 1d outlines the basis of TMM. Following the established methodology by Dai *et. al.*[1], the E-field solution in 2D for each of the media for this stratified system can be written as $\mathbf{E}^j(j,z) = \exp(iqj)\ \xi^j(z)$ where $j = x, y$ and $\mathbf{q}$ is the tangential wavevector and the amplitude function is $\xi_m^j(z) = A_m \exp(-ik_m^z z) + B_m \exp(ik_m^z z)$, where m = 0, 1, 2, 3, 4 represents any of the three media from top to bottom which are respectively air, α-MoO_3, Ge, Au and Si. The boundary conditions require q_m to be constant in all boundaries, therefore,

$$k_m^z = \sqrt{\varepsilon_m^j \left(\frac{\omega^2}{c^2} - \frac{q^2}{\varepsilon_m^{z\,2}}\right)}, \operatorname{Im} k_m^z > 0 \quad \text{(S1)}$$

where c represents the speed of light in vacuum. Due to an oblique incidence angle of θ = 25° in FTIR system, $\mathbf{q}$ (wavevector component parallel to the interfaces) will be nonzero. Since q_m must be fixed through all boundaries, $q_m = q = (\omega/c)\sin(\theta = 25°)$. The coefficients of the amplitude function can be represented in terms of the reflection and transmission coefficients from the boundaries, which can be calculated as

$$Q_m = \frac{\varepsilon_m^j}{k_m^z},\ r_{mn} = -\frac{Q_m - Q_n}{Q_m + Q_n}, \qquad t_{mn} = 1 + r_{mn}, \qquad n = m+1. \tag{S2}$$

Using Eq. (S2), we can establish the recursive matrices,

$$B_{m,n} = \frac{1}{t_{m,n}} \begin{bmatrix} 1 & r_{m,n} \\ r_{m,n} & 1 \end{bmatrix},\ P_{m,n} = \begin{bmatrix} e^{-ik_z^n d_n} & 0 \\ 0 & e^{ik_z^n d_n} \end{bmatrix} \tag{S3}$$

$$\begin{bmatrix} E_0 \\ rE_0 \end{bmatrix} = \prod_{m=0}^{N} T_{m,n} \begin{bmatrix} tE_0 \\ 0 \end{bmatrix},\ T_{m,n} = B_{m,n} P_{m,n} \tag{S4}$$

where d_n is the thickness of the corresponding layer and N is the total number of layers, and matrices B, P and T are respectively the boundary condition, propagation and transmission matrices. It is worth pointing out that in the total matrix the illumination is from top; hence, the second row in the right-hand 2×1 matrix of equation (S4) vanishes. Thus, the total reflectance can be calculated as,

$$\prod_{m=0}^{N} T_{m,n} = \begin{bmatrix} T_{11} & T_{12} \\ T_{21} & T_{22} \end{bmatrix}, \qquad R = |r|^2 = |\frac{T_{21}}{T_{11}}|^2 . \tag{S5}$$

The total absorption then can simply be calculated as $A = 1 - R$ given the fact that the thick bottom reflector blocks any transmission through the multilayer structure. The coded TMM method for total absorption versus wavelength and t_{Ge} when t_{MO} = 0.15 μm for x polarized light (θ = 0°) is illustrated in Figs. 2b and c. The calculated absorption contour of Fig. S1a is carried out using Lumerical FDTD software and is strictly analogous to the TMM result demonstrated in Fig. 2a of the manuscript. The pink dot in Fig. 3a (*xz* cross is the OPh_y peak and is not observed in Fig. 3a which shows the the absorption map in *xz* cross-section (ϕ_i = 0°). The ϕ_i = 90° counterpart is illustrated in Supplementary Figure 1b and is in agreement with the experimental pink dot. Besides, the hybridization of α-MoO_3 FP mode with OPh_y is evident for $\omega < \omega_{TO,y}$.

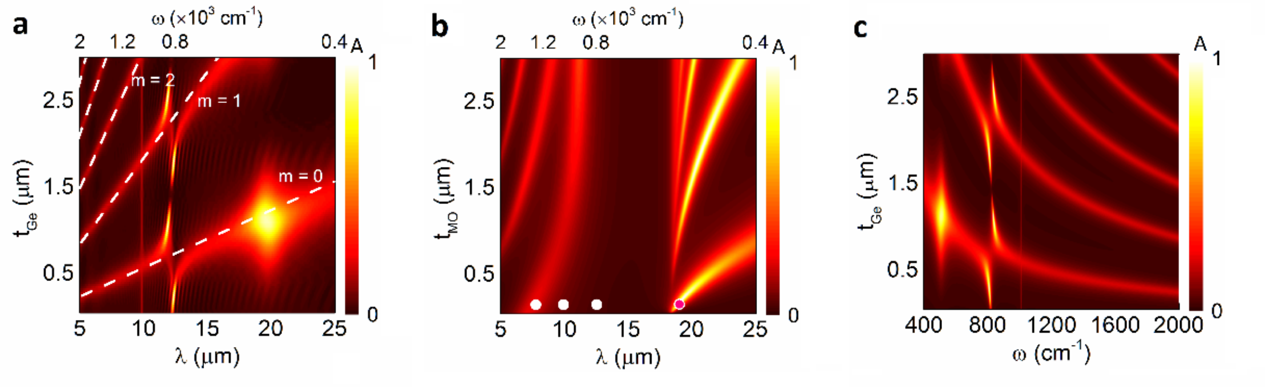

Supplementary Figure 1. (a) Total absorption versus wavelength and t_{Ge} when t_{MO} = 0.15 μm for θ = 0°, (b) absorption versus wavelength/frequency and t_{MO} in the multilayer structure for t_{Ge} = 0.42 μm where ϕ_i = 90°. (c) Total absorption versus frequency and t_{Ge} when t_{MO} = 0.15 μm for ϕ_i = 0°.

The linear dependence of the resonant absorption frequency upon the t_{Ge} can be fit to the Fabry Perot (FP) phase-matching condition. We fit linear FP oblique lines representing the observed FP modes.

Considering the wave trapped within the Ge layer, it reflects back from the top and bottom boundaries of Ge with α-MoO_3 and Au layers respectively. Thus, the FP condition for phase match dictates described by

$$\varphi_{r,MO} + 2k_{Ge}t_{Ge} + \varphi_{r,Au} = 2m\pi, m = 1, 2, 3, ... \tag{S6}$$

Simplifying this equation to linear dependence of t_{Ge} on λ yields $t_{Ge} = (\lambda/2n_{Ge})(m - (1/2\pi)(\phi_{r,MO} + \phi_{r,Au}))$, m = 1,2,3,…, where n_{Ge} is the refractive index of Ge, $\phi_{r,MO}$ and $\phi_{r,Au}$ are the phases picked up upon reflection from the interfaces of Ge, with α-MoO_3 and Au layers, respectively. Fitting the FP condition to the fundamental mode (m = 1) and using n_{Ge} = 4 yields $\phi_{r,total} = \phi_{r,Au} + \phi_{r,MO}$ = 167° [42]. Applying the same fitting procedure to the next order mode, m = 2, results in $\phi_{r,total}$ = 164°, which is close to the previous value and justifies our description of this as FP behavior. The next higher-order modes follow similar patterns and correspond to the modes m = 3, 4. This result highlights the existence of FP modes through the linear dependence of t_{Ge} on λ and are summarized in Supplementary Table 1. Since frequency is inversely related to wavelength, the resonant absorption versus frequency in cm^{-1} will yield nonlinear dependence. Supplementary Figure 1c illustrates total absorption in the multilayer FP structure shown in Fig. 1d with the same parameters used to calculate Supplementary Figure 1a to underline this contrast. As a result, the FP condition yields

$$t_{Ge} = \frac{1}{2\omega n_{Ge}}(m - \frac{\varphi_{r,MO} + \varphi_{r,Au}}{2\pi}) \tag{S7}$$

which infers nonlinear dependence of t_{Ge} on ω. The $t_{Ge} \propto 1/\omega$ dependence is evident in Supplementary Figure 1c contour plot. FTIR measurements were also carried out on samples with two t_{Ge} values which are used for samples S1, S2 and S3. These results together with TMM calculated spectra are illustrated in Supplementary Figure 2b and c and are reasonably in agreement with each other.

Mode number	Δt_{Ge} (μm)	$\Delta\lambda$ (μm)	$\phi_{r,tot}$ (deg)
m = 1	1.342	20	166.72
m = 2	2.043	10.58	163.87
m = 3	1.489	4.71	169.07
m = 4	0.87	1.975	170.97

Supplementary Table 1. Linear fit values for the FP modes in Ge layer sandwiched between α-MoO_3 and Au.

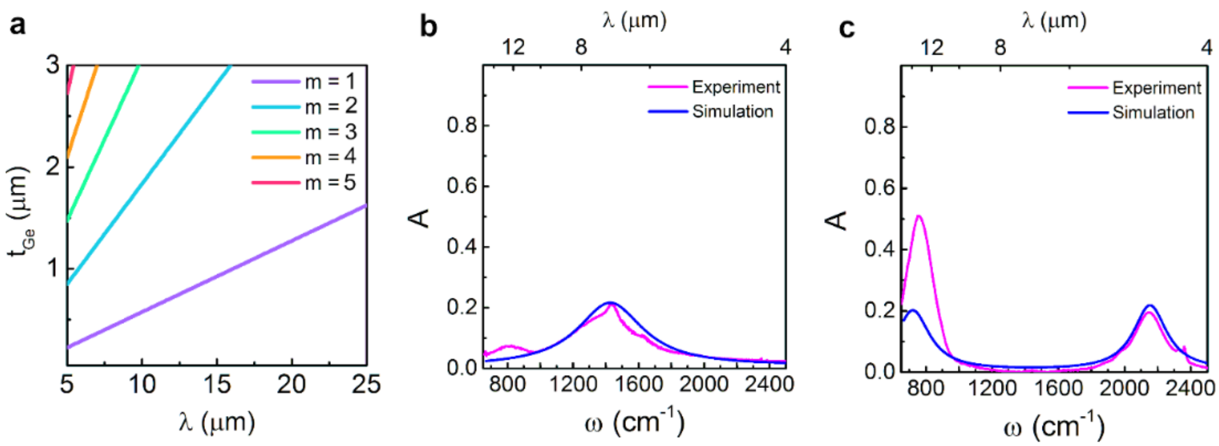

Supplementary Figure 2. (a) Linear fit to the FP modes of Ge cavity. Spectral absorption in multilayer structures without α-MoO_3, composed of Au and Ge with (b) t_{Ge} = 0.42 μm and (c) t_{Ge} = 0.85 μm. In panels b and c, the solid and dashed lines represent the experimental and TMM calculated results, respectively.

Note 2. α-MoO_3 flakes.

Raman spectra of the layered α-MoO_3 flakes used for this study are provided in Supplementary Figure 3 and the OPh_z, OPh_x and OPh_y are labeled respectively with A_g, B_{1g} and B_{3g}.

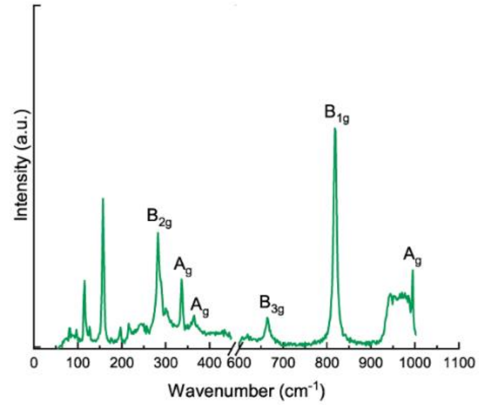

Supplementary Figure 3. Raman spectra of layered α-MoO_3 flakes. A_g, B_{1g}, B_{2g}, and B_{3g} phonon modes are labeled.

The optical images of all the flakes discussed in the main body are presented here. The optical images of the samples (flakes) S1, S2 and S3 are illustrated in Supplementary Figure 4. As mentioned in the main body, the roughness of these flakes is quite large. In fact, the larger flakes are also the ones with more extreme roughness values. Since large flakes are necessary to have acceptable signal to noise ratio in mid-IR for FTIR characterization, we conducted the study on larger flakes. The variations in color in the optical images of Supplementary Figure 4 also infer the rough surface of the flakes. SEM images of Supplementary Figures 4d-f and surface profilometer scans of Supplementary Figures 4g-i shed light on the surface roughness of the three flakes. The major consequences of rough surface are the observation of enhanced

OPh_z, resonance decrease and broadening of the OPh_x and OPh_y peaks and the suppression of the OPh_y mode.

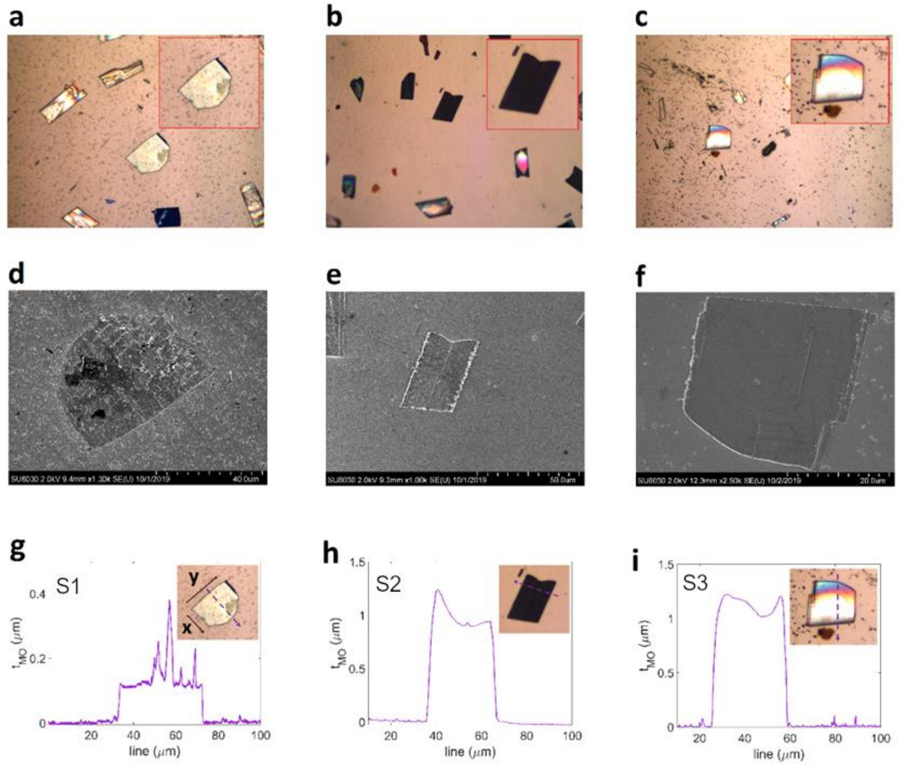

Supplementary Figure 4. Optical images of flakes (a) M1La, (b) M1Lb and (c) M2Lc. (d)-(f) SEM images and (g)-(i) surface profile of the flakes in the same order.

In order to determine the crystal directions of the α-MoO_3 flakes, the flakes were measured with FTIR with a built-in linear polarizer in the optical path of the incident radiation. Revolutions of 6.5° were used to find the maximum peak in 820 cm^{-1} which outlines OPh_x. The orthogonal direction to *x* would be the designated *y* direction. The overlaid FTIR response of the S1, S2 and S3 samples and their simulated counterparts are represented in Supplementary Figures 5a-c and the experimental offset plots with 22.5° revolution are provided in Fig. 4 in the manuscript.

As hinted in the manuscript, the peak locations of the experimental results of Fig. 4 are in agreement with the simulation results of Figs. 2 and 3. However, there are discrepancies between the experiments and simulations as is evident from Supplementary Figures 5. The most fundamental deviation occurs due to the roughness of rather thick (400 and 800 nm) PVD deposited Ge. Another origin of discrepancy is attributed to the flake roughness as depicted in Supplementary Figures 4. In the simulations, we assume single average thickness values for Ge and α-MoO_3 for simplicity, all of which are mentioned in the caption of Fig. 4 (also in Supplementary Figures 5). Since the thicknesses of the flake and Ge are not uniform over the measurement area, the peaks are broadened in the expriments. Moreover, as mentioned in the manuscript, since OPh_y occurs at wavelength values beyond 18 μm, the flake area should be smooth enough over comparable lengths in order to observe OPh_y in the FTIR reflectance signal clearly. However, the roughness of the flakes hinder long-wave signal detection in reflectance mode off of the flakes.

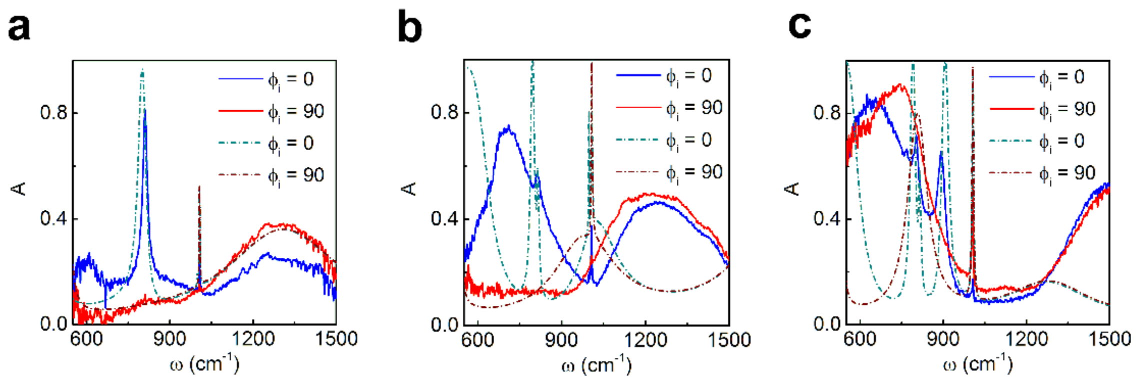

Supplementary Figure 5. Polarization-dependent absorption spectra of fabricated samples, schematically illustrated in Fig. 1d with thick Au, (a) S1, t_{Ge} = 0.42 μm and t_{MO} = 0.15 μm, (b) S2, t_{Ge} = 0.42 μm and t_{MO} = 0.95 μm and (c) S3, t_{Ge} = 0.85 μm and t_{MO} = 1.1 μm. Incident polarization (ϕ_i) is set to *x*-direction (0°) and *y*-direction (180°). The dashed lines are the simulations results.

Note 3. Birefringence.

The main results represented in Fig. 5 of the manuscript justify polarization rotation when ϕ_i = 45° and ϕ_a = -45°. This stems from the fact that the incident polarization is neither along *x* nor *y* directions. Therefore, having nonzero initial values in both in-plane directions (ϕ_i = 45°) results in the observed birefringence due to the fact that the decomposed light to the two directions experience different dielectric functions. Contrarily, if the incident polarization is set to be parallel to any of the crystal directions, no polarization rotation should be detected to cross-polarization after the analyzer. This experiment is carried out on the same sample (S2) and the result is illustrated in Supplementary Figure 6a. In this experiment, ϕ_i = 0° (parallel to *x*) and in the case of ϕ_a = -90° (parallel to *y*, pink curve), as expected, no substantial intensity is detected. Supplementary Figure 6b also shows the reflectance of the substrate (Au-Ge) for ϕ_i = 45°, while ϕ_a = 45° and ϕ_a = -45°, which is the same condition for the result of Fig. 5 in the manuscript. Since Au-Ge layers are isotropic, no cross-polarized reflectance signal is detected (pink curve). Besides, the green curve in Fig. S6b is used as reference to correct the attenuated detection signal due to the contribution of two polarizers (ϕ_i and ϕ_a) that render the total transmittance to less than 100%.

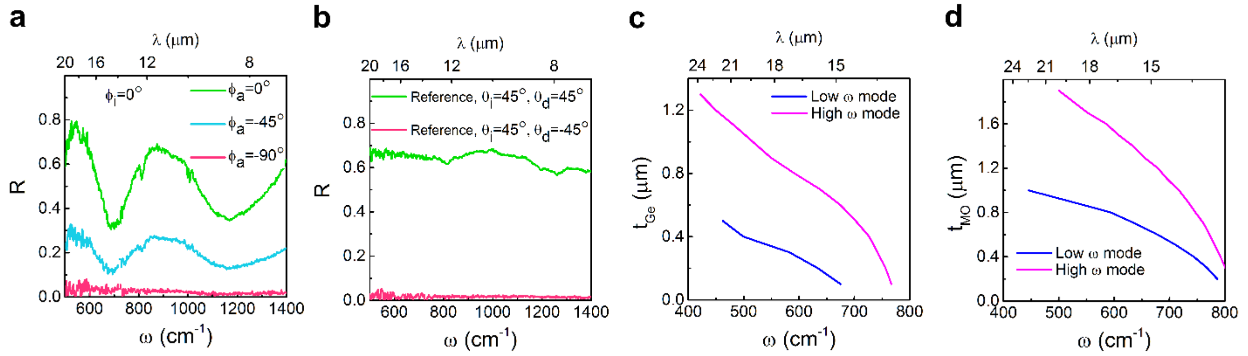

Supplementary Figure 6. (a) Reflectance of sample S2 when ϕ_i = 0°, while ϕ_a = 45° and ϕ_a = -90°. (b) Reflectance of Au-Ge substrate (no flake) when ϕ_i = 45° and ϕ_a = -45°. Tracing the two modes of circularly polarized reflectance frequency when (c) t_{MO} = 0.95 μm and t_{Ge} is changed and (d) t_{Ge} = 0.42 μm and t_{MO} is swept.

The circular polarization can be tuned with device parameters. From Fig. 5c in the manuscript, it is inferred that for S2 sample, we have two frequencies that circularly polarized reflectance is taking place. However, due to reduced SNR in low frequency limit, the resolution of experiment does not permit for reflectance signals in the order of 10% to be recorded clearly. As a result, we observe one peak experimentally, around 730 cm^{-1}. Supplementary Figure 6c demonstrates the effect of modifying t_{Ge} (while keeping t_{MO} = 0.95 μm) on the frequency of circularly polarized light can be tuned for both low and high frequency circular polarization points. Figure S6d illustrates the effect of modifying t_{MO} (while keeping t_{Ge} = 0.42 μm) on the frequency of circularly polarized reflected signal. The FDTD simulation results of Supplementary Figure 6c and d are also validated by TMM calculations as demonstrated in Fig. 5f and h, respectively.

The simulated polarization ellipse at 730 cm^{-1} is illustrated in Fig. 5d. More examples of sample S2 polarization ellipse, where ϕ_i = 45° and ϕ_a = -45° (identical to the results of Fig. 5d) at four frequency values less and greater than 730 cm^{-1} is illustrated in Supplementary Figure 7 which shows the transformation of polarization ellipse from almost parallel to -45° to 45°. Specifically, two polarization values at 700 cm^{-1} (Supplementary Figure 7b) and 750 cm^{-1} (Supplementary Figure 7c) imply that the significant rotation of polarization ellipse of approximately 90° is taking place between these two values. This is indeed true and as a result, circularly polarized reflected light is expected at 727 cm^{-1}. Contrarily, at 650 cm^{-1} a linearly polarized light is obtained.

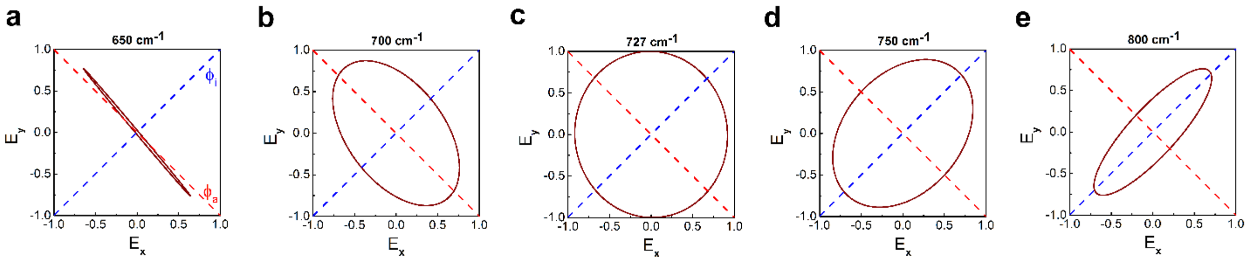

Supplementary Figure 7. Simulated reflected polarization ellipse where ϕ_i = 45 and ϕ_a = -45 at (a) 650 cm^{-1}, (b) 700 cm^{-1}, (c) 727 cm^{-1}, (d) 750 cm^{-1} and (e) 800 cm^{-1}.

In order to highlight the advantage of the FP structure compared to a single flake (on a Ge substrate, for the sake of consistency and feasibility), the Q_{circ} simulations are repeated for α-MoO_3-Ge structure as follows.

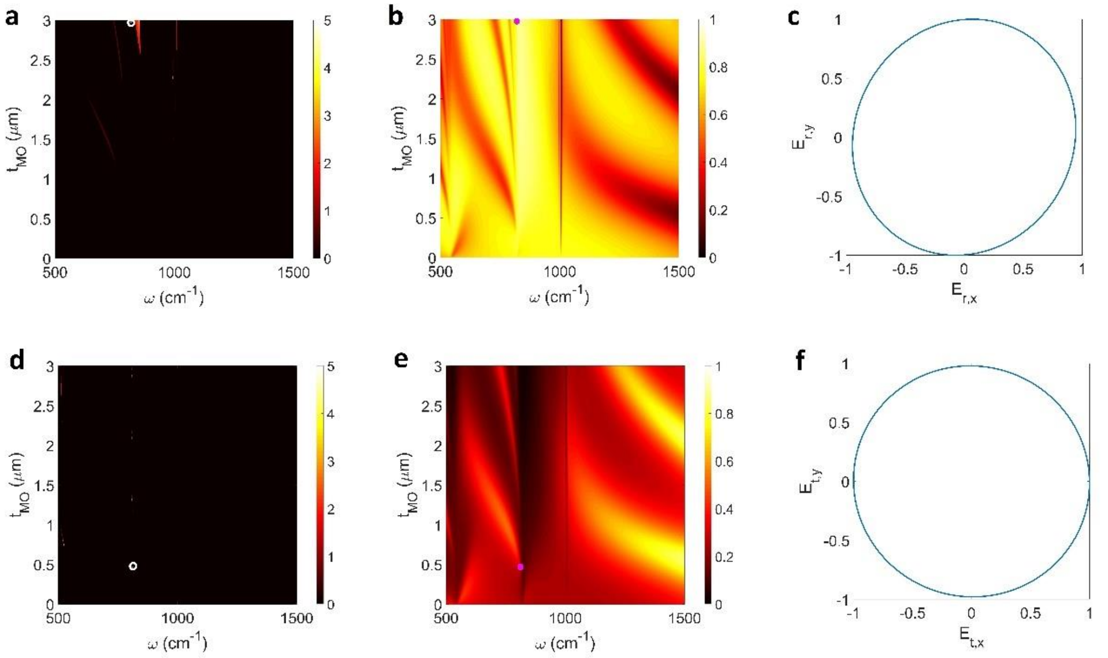

Supplementary Figure 8. (a) Simulated spectral quarter-wave plate action quality factor versus t_{MO}, (b) spectral reflectance versus t_{MO} and (c) simulated polarization ellipse of reflected beam at ω = 820 cm^{-1} and t_{MO} = 3 μm, all in reflection mode with θ = 25° and ϕ_i = 45°. (d) Simulated spectral quarter-wave plate action quality factor versus t_{MO}, (e) spectral transmittance versus t_{MO} and (f) simulated polarization ellipse of transmitted beam at ω = 815 cm^{-1},and t_{MO} = 0.465 μm, all in transmission mode with θ = 25° and ϕ_i = 45°. The dots in (a-b) and (d-e) represent the data points for an example of polarization ellipse respectively demonstrated for (c) reflection and (g) transmission.

As discussed in the manuscript, the existence of transmission in the absence of Au layer (α-MoO_3-Ge) makes it more difficult for the circular polarization condition to be satisfied, specifically for the amplitude condition. As a result, there are less accessible frequency values for quarter-wave plate action as inferred from Supplementary Figure 8a and d which sketch Q_{circ} as a function of frequency and α-MoO_3 thickness. Another disadvantage of not using the FP structure is lower reflectance and the flow of some proportion of the power to transmittance, presented respectively in Supplementary Figures 8b and e. Supplementary Figure 8c shows an example of the polarization ellipse in reflection mode from a 3 μm α-MoO_3 on Ge substrate at 820 cm^{-1} (this data point is highlighted with dots on Supplementary Figures 8a and b). The reflectance for this circularly polarized case is 79% (highest reflectance, shown with purple dot on Fig. S8b) that is 15% less than most of the results for the FP structure with Au. The example designated for transmission mode is illustrated in Supplementary Figure 8f. This panel shows the polarization ellipse in transmittance mode from a 0.465 μm α-MoO_3 on Ge substrate at 815 cm^{-1} and the transmittance is 19%; thus, the efficiency in transmission mode is far less than reflection counterpart as well as the FP results put forth in the manuscript.

The last parameter that can be modified to tune the polarization state of radiation is the incident polarization. In all of the polarization simulations, $\phi_i = 45°$. By changing ϕ_i, the circularly polarized reflection frequency can be slightly tuned in FP structure as demonstrated in Supplementary Figure 9.

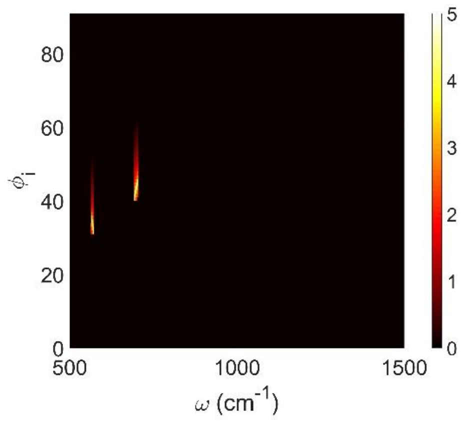

Supplementary Figure 9. Simulated spectral quarter-wave plate action quality factor versus ϕ_i, where $t_{MO} = 0.95$ μm, $t_{Ge} = 0.42$ μm and $\theta = 25°$.

References.

1. Dai, S. *et. al*. Graphene on hexagonal boron nitride as a tunable hyperbolic metamaterial. *Nat. Nanotechnol.* **10**, 682 (2015).
